# Draft genome sequence of the plant growth-promoting rhizobacteria *Streptomyces* sp. CLV115

**DOI:** 10.1128/mra.00827-24

**Published:** 2024-12-10

**Authors:** Natasha Ruschel Soares, Juan Felipe Fernández Campos, Luiza Zardin Missau, Thiago Rosa Pianetti, Júlia Budelon Gonçalves, Eliane Romanato Santarém, Leandro Vieira Astarita

**Affiliations:** 1 PUCRS, Laboratory of Plant Biotechnology, Porto Alegre, Rio Grande do Sul, Brazil; University of Maryland School of Medicine, Baltimore, Maryland, USA

**Keywords:** genomic, *Streptomyces*, PGPR, rhizosphere, secondary metabolism

## Abstract

*Streptomyces* sp. CLV115 isolated from Solanaceae rhizosphere in southern Brazil shows promising plant growth-promoting traits; thus, whole-genome sequencing aids the unraveling of its genomic potential, offering insights into developing natural crop solutions and advancing *Streptomyces* genomics.

## ANNOUNCEMENT

*Streptomyces,* the largest genus of actinomycetes, is known for producing a wide array of bioactive molecules, with pharmaceutical, industrial, and agricultural relevance (1). These bacteria are rhizosphere colonizers, using their diverse metabolome to thrive in competitive environments (2). *Streptomyces* protect the root systems from pathogens by secreting toxic compounds while influencing plant development by synthesizing growth regulators, traits typical from plant growth-promoting rhizobacteria, and pose as candidates for biocontrol and biofertilizer formulations (3). Advancing research toward their rich genomic potential is essential for developing natural crop solutions. In the “omics” era, whole-genome sequencing emerges as a necessary tool to unravel essential features embedded within genomic sequences.

Rhizospheric soil was collected from *Solanum pseudocapsicum* L. in Porto Alegre (−30.084.715; −51.129.550; Brazil). Bacterial isolation was performed on ISP2 (International Streptomyces Project-2) medium supplemented with antimicrobial agents (4). Bacterial genomic DNA was extracted using the Wizard Genomic DNA Purification Kit (Promega, USA). PCR amplification of the 16S rRNA gene was performed using the universal primers 9F (5′-AGAGTTTGATCCTGGCTCAG-3′) and 1542R (5′-AGAAAGGAGGTGATCCAGCC-3′) and the amplicons sequenced by ACTGene Análises Moleculares (Brazil) using two sets of primers: 9 F-1542R and MG3F (5′-CAGCAGCCGCGGTAATAC-3′) and 800R (5′-TACCAGGGTATGTAATCC-3′). The analysis confirmed the strain belongs to the *Streptomyces* genus (GenBank accession number ON723951.1). Whole-genome sequencing represents the logical next step to further investigate genomic potential not yet detected in experiments. *Streptomyces* sp. CLV115 cells were cultured on ISP2 agar medium at 28°C to obtain isolated colonies, and samples were sent to GoGenetic (Curitiba, PR–Brazil) for DNA extraction (Qiagen QIAmp DNA kit), genomic library construction (Nextera XT Sample Preparation Kit, Illumina), and whole-genome sequencing (MiSeq System, Illumina). Trimmomatic v.0.39 (5) was used to remove low-quality regions and adaptor sequences. Reads were assembled using SPAdes v.3.15.4 (6). Sequence quality was assessed using QUAST v.4.4 (7) and CheckM 1.0.18 (8).

Prokka v.1.14.6 (9) was used for automatic genomic sequence annotation. Overall sequencing statistics are provided in Table 1. Default parameters were used except where otherwise noted.

**TABLE 1:** Sequencing statistics for whole-genome sequencing of *Streptomyces* sp. CLV115

Organism	*Streptomyces* sp. CLV115
# contigs	483
Biggest contig size (bp)	180,742
Genome size (bp)	9,468,001
GC (%)	70.86
# reads	4420709
N50 (bp)	48,523
Coverage	119.4
Illumina read length	Paired end 2 × 300 bp

The genomic sequence for *Streptomyces* sp. CLV115 (RefSeq GCF_038448545.1) comprises 9,468,001 bp (G + C content of 70.86%) (Table 1). Annotation revealed a total of 8,386 predicted genes, including 8,271 protein-coding sequences and 77 tRNAs. *Streptomyces gelaticus* (91.3% ANI (Average Nucleotide Identity); GenBank accession [GCA_014649535.1]) was identified as the closest genome available, according to MiGA (Microbial Genomes Atlas). By utilizing AntiSMASH 7.0 (10), 43 biosynthetic clusters were identified, 28 of which could be matched to clusters from other *Streptomyces* spp. BlastKOALA (11), a KEGG (Kyoto Encyclopedia of Genes and Genomes) tool for functional characterization, was used to explore genome annotation. The database analysis revealed over 500 coding sequences dedicated to secondary metabolism and environment adaptation. These findings corroborate the largeness and diversity of *Streptomyces* genomes compared to most bacteria, reflecting the significant effort that streptomycetes devote to the production of secondary metabolites.

## Data Availability

This Whole Genome Shotgun project has been deposited in DDBJ/ENA/GenBank under the accession number GCA_038448545.1 (raw reads are under SRA accession number SRR28746463).
